# How Are Consensual, Non-Consensual, and Pressured Sexting Linked to Depression and Self-Harm? The Moderating Effects of Demographic Variables

**DOI:** 10.3390/ijerph18052597

**Published:** 2021-03-05

**Authors:** Sebastian Wachs, Michelle F. Wright, Manuel Gámez-Guadix, Nicola Döring

**Affiliations:** 1 Department of Educational Studies, University of Potsdam, 14476 Potsdam, Germany; wachs@uni-potsdam.de; 2 National Anti-Bullying Research and Resource Centre, Dublin City University, C109 Dublin, Ireland; 3 Department of Psychology, Pennsylvania State University, University Park, PA 16802, USA; 4 Department of Psychology, DePaul University, 1 E. Jackson, Chicago, IL 60604, USA; 5 Department of Biological and Health Psychology, Autonomous University of Madrid, 28049 Madrid, Spain; manuel.gamez@uam.es; 6 Department of Media and Communication Science, Ilmenau University of Technology, 98693 Ilmenau, Germany; nicola.doering@tu-ilmenau.de

**Keywords:** sexting, depression, self-harm, non-consensual, pressured sexting, normalcy discourse

## Abstract

Sexting among adolescents has triggered controversial debates among scholars and the general public. However, questions regarding the associations between different types of sexting, namely consensual, non-consensual, and pressured sexting, depressive symptoms, and non-suicidal self-harm remain. In addition, little attention has been given to whether demographic variables (i.e., gender, ethnicity, disability, sexual minority) might influence these associations. To fill these gaps in the literature, the present study was conducted. Participants were 2506 adolescents (ages 13–16 years old; M_age_ = 15.17; SD_age_ = 0.89) from eight high schools located in the suburbs of a large Midwestern city in the United States. Adolescents self-identified as female (50%), Caucasian (57%), approximately 15% reported that they had a disability they received school accommodation for, and 18% self-identified as a sexual minority. They completed self-report questionnaires on their sexting behaviors, depressive symptoms, and non-suicidal self-harm. Findings revealed that non-consensual and pressured sexting were positively related to depressive symptoms and non-suicidal self-harm, whereas consensual sexting was unrelated to these outcomes. Boys engaged in more non-consensual sexting compared with girls, girls were more pressured to send sexts compared with boys, and sexual minority adolescents reported greater consensual sexting compared with non-sexual minority adolescents. Moderating effects revealed that girls, non-minority adolescents, and non-sexual minority adolescents experienced greater depressive symptoms and non-suicidal self-harm when they experienced pressured sexting. These findings underscore the importance of considering various types of sexting and adolescents’ demographic variables when examining the negative outcomes of sexting. Disentangling the relationships among different types of sexting, depressive symptoms, and self-harm aids in the development of evidence-based recommendations for sexting harm prevention and sexual education programs.

## 1. Introduction

For adolescents, the use of information and communication technologies (ICTs) is an integral part of their daily lives [1]. ICTs have become powerful tools for education, identity formation, entertainment, managing/maintaining romantic relationships, and sexual exploration and expression [2,3]. A contemporary form of sexual online expression is sexting which is defined as sharing sexually suggestive text messages or self-produced sexually explicit images like photos or videos (sexts) via social media or smartphone apps [4,5,6]. With increasing research on sexting, scholars have identified various types of sexting, leading to the understanding that consensual sexting might likely be risky but not an overall problem and is a normal form of contemporary sexual communication, whereas other types of sexting, which involve pressure, blackmailing, or the unasked forwarding of sexts, are considered as harmful with severe consequences [7,8,9]. Additionally, yet, little research attention has been given to varying associations among different types of sexting behaviors, namely consensual, non-consensual, and pressured sexting, and negative psychological outcomes, and whether demographic variables (i.e., gender, ethnicity, disability, sexual minority) might influence these associations. Furthermore, while early adolescents between 12 and 15 years are particularly vulnerable for negative outcomes, this age group is largely under-researched [10].

Although students are interested in learning more about how they can practice safer sexting, schools rarely include the topic of sexting in their sex education curriculum [11]. If schools do include sexting in this curriculum, sexting is often portrayed as inherently negative and sexting abstinence is recommended without reflecting on either different sexting types or recommendations for safer sexting practices [11,12]. The present study has been conducted to equip adolescents, parents, guardians, educators, and teachers with evidence-based recommendations, inform the development of sexting harm prevention programs, and close the gaps in the research literature.

### 1.1. Sexting Discourses and Different Types of Sexting

The developmental significance and potential risk of sexting behaviors among adolescents have been hotly debated, resulting in two different perspectives [4]. One side of the debate views sexting as a normal contemporary expression of sexual behavior that addresses developmental needs and tasks and is often carried out within both casual and committed romantic relationships [9,13,14,15,16,17,18]. From this perspective, sexting is viewed as a mechanism to explore sexuality; develop sexual identity; increase fun, intimacy, and passion among partners; and as facilitating communication among minority groups [4,19]. Confirming the normalcy perspective of sexting are studies that revealed (1) the relatively high prevalence of sexting in different population groups, and (2) the relatively low prevalence of sexting harms [15]. The second side of the debate frames sexting as dysfunctional and deviant behavior which at least increases adolescents’ exposure to or involvement in more risky behavior, such as substance use, risky sexual behaviors (e.g., promiscuity, unprotected sexual intercourse), and other online risks (e.g., cyberbullying, cybergrooming, and sexual online solicitation by adults [8,20,21,22,23,24,25,26]).

Although most earlier studies on sexting among adolescents employed a deviance perspective [4], current evidence increasingly supports the normalcy discourse. An important next step is the operationalization of sexting as a multidimensional construct reflecting different types of sexting. When sexting is performed voluntarily in the absence of pressure and blackmailing and the sexts are not forwarded without the consent of the person who produced the sexts, most scholars refer to it as *consensual sexting* [4,27]. *Aggravated sexting*, on the other hand, involves the presence of harmful intention toward someone who shares sexts or forces someone to share sexts [28]. This form of sexting involves two different types including non-consensual sexting and pressured sexting. The sharing of sexts without permission is referred to as *non-consensual sexting* [27]. Sometimes sexting is the result of pressure by a partner or friend to send sexts, referred to as *pressured sexting* [27,29,30].

### 1.2. Links between Sexting and Depression and Non-suicidal Self-harm

The associations between sexting and psychological problems have attracted some research attention. Many of the studies on this topic are cross-sectional and it remains unclear whether psychological problems might be antecedents or consequences of sexting or even both. On the one hand, several authors argue that adolescents who show psychological problems (i.e., depressive symptoms, anxiety, self-harm) participate in sexting to feel considered and desired, and they conclude that those adolescents might have fewer coping strategies to deal with the social pressure to sext. In addition, psychological difficulties might impair adolescents’ decision-making processes and risk assessment and thus, those adolescents might underestimate potential risks associated with sending (semi-)nude pictures of oneself [31,32]. On the other hand, psychological problems might be a consequence of aggravated sexting. Forwarding sexual content without consent can damage the victim’s reputation and decrease emotional well-being [12,33]. This assumption is also supported by research showing that often the victims but not the perpetrators of aggravated sexting are blamed by peers and adults for the unwanted dissemination of sexually explicit pictures [34,35]. Victims of aggravated sexting might feel stressed by the feeling that they cannot control the situation and feel helpless. These negative feelings can manifest in psychological difficulties (e.g., depression, anxiety; [36]) and might explain why victims of aggravated sexting might show a higher risk for psychological difficulties.

Some research has investigated the association between sexting and depression, revealing inconsistent findings. While several studies found a positive association between depressive symptoms and sexting [5,31,33,37,38,39,40,41,42], other studies showed no significant relationship [27,43,44,45], and yet other investigations revealed mixed results [32,46,47]. For example, Temple et al. [46] found in a sample of 938 adolescents between 14- and 18-years-old that the link between depression and sexting became insignificant after including sexual behavior, age, gender, race/ethnicity, and parent education as control variables. Klettke et al. [32] showed in a study with 589 participants aged 17- to 21-years-old that depression was only significantly correlated with sexting among male and not female participants. Conversely, Ybarra and Mitchell [47] found in a sample of 3715 adolescents between 13- and 18-years-old that only for female but not male participants depressive symptoms were related to sexting. Potential reasons for these inconsistent findings on the relationship between sexting and depressive symptoms are differences in sample characteristics (i.e., age, gender distribution) and the operationalization of sexting (i.e., global items, one or two types of sexting). Several studies used global items without specifying whether sexting was conducted consensually, non-consensually, or under pressure [32,33,44,45,46], whilst other authors measured one or two of the three types of sexting [31,38,39,41,43,47]. Only one of the reviewed studies [27] considered all three types of sexting. However, this study with 1334 participants between 13- and 30-years-old did not analyze the relationship between depression and sexting by considering the three different types separately but investigated sexting groups according to their frequency of involvement (low, moderate, and high) across all three types of sexting. The findings suggest that moderate/high sexters did not show a higher risk for psychological difficulties (e.g., depression, anxiety) but they did for dating violence [27].

Another potential consequence of aggravated sexting behavior might be non-suicidal self-harm. Non-suicidal self-harm is defined as deliberate, self-injurious behavior without suicidal intent [48]. Research that investigated the relationship between sexting behavior and self-harm is scarce. In one study with 617 college students, pressured sexters reported more digital self-harm (self-cyberbullying) compared with non-pressured sexters or non-sexters [38]. In another study with 6021 9th to 12th graders, both consensual and non-consensual sexting were positively related with self-harm [39]. Yet another study with 1372 participants between 15 and 29 years old showed that sexting was associated with alcohol consumption which resulted in either injury to self or others [18]. None of these studies considered consensual, pressured, and non-consensual sexting in one analysis.

Clearly, more research is needed to better understand whether there are differences in the impact of consensual sexting, non-consensual sexting, and pressured sexting on adolescents’ depressive symptoms and non-suicidal self-harm. It is also unclear how demographic variables might increase adolescents’ risk of depressive symptoms or non-suicidal self-harm resulting from different types of sexting behaviors.

### 1.3. Moderating Effects of Demographic Variables

Researchers have considered the risk factors associated with sexting among adolescents, including demographic variables that increase risk, such as gender, ethnicity, disability, and sexual minority status. Studies on gender differences in sexting are mixed, with some revealing that boys engaged in sexting more so than girls and yet others indicated that girls engaged in sexting more than boys [30,31,40,47,49,50,51,52,53]. Some research has found that boys forward and request sexual photos more than girls and girls indicate that they are asked for such content more often than boys [54,55]. Other research has revealed no gender differences in sexting [8,24,56,57,58]. Conclusions from a meta-analysis suggested no gender differences in sexting behaviors [8]. However, gender might increase the risk of negative outcomes associated with sexting. Girls might be more vulnerable to the negative outcomes of sexting because sexting might be more damaging to their reputation, partially explained through widespread sexual double standards [35,47,59].

Some attention has been given to racial and ethnic minority differences in sexting behaviors. Racial and ethnic minority adolescents engaged in more sexting than non-ethnic minority adolescents [37]. Another study revealed that Asian/Pacific Islanders were five times more likely to be sexters than other ethnic minority groups [44]. Yet, a different study with white college students revealed that this group was more likely to engage in sexting than non-white college students [20]. Therefore, conclusions regarding risk based on ethnic minority status are unclear. It is further unclear how ethnic minority status might alter the relationship between different types of sexting behaviors and negative outcomes, such as depressive symptoms and non-suicidal self-harm. Differences in sexting frequencies and behaviors between ethnic minorities and white adolescents might be explained by stressors induced by discrimination and victimization [60].

Little attention has been given to whether disability status influences sexting behaviors and negative outcomes. Oftentimes, the sexuality of disabled adolescents is minimized or ignored, with the belief that these individuals are asexual or hypersexual, unable to control urges [61,62]. Ultimately, the research indicates that adolescents with disabilities express positive desires, attitudes, and expectations concerning future sexual relationships [63]. Adolescents with disabilities report greater consensual and forced sex than their nondisabled peers [64]. Considering that disabled adolescents have sexual desires and act on those desires, it is important to understand their experiences of sexting behaviors by examining differences in frequency of these behaviors versus nondisabled peers and associated negative outcomes. Research on disabled adolescents’ experiences of sexting is important to inform intervention programs sensitive to the needs of diverse groups of adolescents.

Research has revealed that sexting is much more likely to occur among sexual minority adolescents [14,31,65]. Furthermore, Van Ouytsel et al. [65] found that sexual minority adolescents were more likely to have sent, received, and asked for sexual images when compared to non-sexual minority adolescents, as well as experienced greater pressure to send sexually explicit pictures. However, these adolescents did not perpetrate non-consensual forms of sexting more often than non-sexual minority adolescents. To explain such differences, researchers propose that the online environment protects against sexual stigma and prejudice and provides a safer way for sexual minority adolescents to communicate with each other [19]. It is unclear whether sexual minority adolescents might be at increased risk of the negative outcomes associated with sexting when compared to non-sexual minority adolescents.

### 1.4. Present Study

The aims of the present study were twofold. The first aim was to investigate the associations between three types of sexting behaviors (i.e., consensual, non-consensual, pressured sexting) and depressive symptoms and non-suicidal self-harm. The second aim was to examine the potential moderating role of demographic variables (i.e., gender, ethnicity, disability, sexual minority) in the relationships among various types of sexting behaviors and depressive symptoms and non-suicidal self-harm. The following research questions were developed to guide the present study:

**Research Question 1 (RQ1).**  *How are consensual, non-consensual, and pressured sexting linked to depressive symptoms and non-suicidal self-harm?*

**Research Question 2 (RQ2).**  *How do demographic variables (gender, ethnicity, disability, sexual minority) moderate the relationships between different sexting types and depressive symptoms and non-suicidal self-harm?*

These findings might help to understand more about whether there are differential impacts of different types of sexting behavior and whether these relationships can be explained by further variables. Such information is important for informing the creation of intervention programs that might reduce risks and negative outcomes of sexting and help to identify adolescents based on the type of sexting behavior and pair these adolescents with appropriate treatment.

## 2. Materials and Methods

### 2.1. Participants

The convenience sample of the present study consists of 2506 adolescents (ages 13–16-years-old; M_age_ = 15.17; SD_age_ = 0.89; 50% female) from eight high schools located in the suburbs of a large Midwestern city in the United States. Most adolescents self-identified as Caucasian (57%), followed by Latinx (26%), Black/African American (15%), and Asian (2%). Approximately 15% of adolescents were classified as having a disability. Of the 2506 adolescents, 441 (roughly 18%) indicated that they were a sexual minority (e.g., lesbian, gay, bisexual, questioning, or other) and 2065 self-identified as straight. Schools were in middle-class neighborhoods. No income data were collected from adolescents or their parents, but approximately 30% to 49% of students from the schools qualified for free or reduced cost lunch.

### 2.2. Measures

**Consensual sexting:** Three items were used to assess consensual sexting behaviors [66]. Adolescents were asked to select how many times within the past 30 days they engaged voluntarily in a target behavior with a partner or someone they were interested in using a rating scale of 1 = 0 days, 2 = 1–4 days, 3 = 5–8 days, 4 = 9–12 days, 5 = 13–16 days, 6 = 17–20 days, and 7 = 21+ days. Items included: “How often have you engaged in a sexually suggestive or flirtatious conversation via text”, “How often have you used your phone to send a sexually explicit or provocative image or message”, and “How often have you used your phone to send a nude of nearly nude photo of yourself”. Items were averaged to form a final score of consensual sexting. Higher scores indicate higher consensual sexting. Cronbach’s alpha was 0.86.

**Non-consensual sexting**: The six items from the Non-Consensual Sexting Subscale of the Sexting Questionnaire were used [27]. These items involve privately sending and publicly posting sexts of someone else (e.g., partner, someone they were interested in, an acquaintance) without his/her consent. Sample items include: “How often have you sent sexually suggestive or provocative photos/videos about your partner by SMS/MMS/WhatsApp/Snapchat without his/her consent?”, “How often have you sent sexually suggestive or provocative photos/videos about someone you know by SMS/MMS/WhatsApp/Snapchat without his/her consent?”, “How often have you sent sexually suggestive or provocative photos/videos about your partner over the internet (i.e., Facebook, e-mail, Twitter) without his/her consent?”, “How often have you sent sexually suggestive or provocative photos/videos about someone you know over the internet (i.e., Facebook, e-mail, Twitter)?”, “How often have you publicly posted sexually aggressive or provocative photos about your partner on Facebook, Twitter, or Myspace without his/her consent?”, and “How often have you publicly posted sexually aggressive or provocative photos about someone you know on Facebook, Twitter, or Myspace in without his/her consent?”. Items were rated on a scale of 1 (*Never*) to 5 (*Always or almost daily*). The six items were averaged to form a final score, with higher scores indicating greater non-consensual sexting. Cronbach’s alpha was 0.90 for non-consensual sexting.

**Pressured sexting**: There were two items used to measure perceived pressure by a partner or friends to engage in sexting [27]. The items included: “Sometimes I sext because my friends forced me” and “Sometimes I sext because my partner or someone I’m interested in forced me”. Items were rated on a scale of 1 (*Never*) to 5 (*Always or almost daily*). The items were averaged to form a final score on pressured sexting, with higher scores indicating greater pressure. The correlation between the two items was 0.69.

**Depressive symptoms**: Adolescents completed The Center for Epidemiological Studies Depression Scale to assess depressive symptoms within the last two weeks [67]. They rated 20 items (e.g., I felt sad) on a scale of 0 (*Rarely or none of the time*) to 3 (*Most or all of the time*). Items were averaged to form a final score on depressive symptoms, with higher scores indicating greater symptoms. Cronbach’s alpha was 0.88.

**Non-suicidal self-harm**: The Self-Harm Inventory asked adolescents whether they had ever intentionally engaged in specific behaviors without suicidal intent [68]. Adolescents answered 22 items (e.g., hit yourself, prevented wounds from healing) using yes/no responses. Items were summed to form a final score of non-suicidal self-harm, with higher scores indicating greater non-suicidal self-harm. The Kuder–Richardson reliability was 0.87.

**Demographics**: Adolescents reported their gender, ethnicity, disability, and sexual minority status. All surveyed adolescents selected either male which was coded as 0 or female which was coded as 1 as their gender identity; other gender identities were measured but not reported by participants. They also reported their ethnicity. Ethnicity was coded as 0 for minority status and 1 for Caucasian. Adolescents were asked about whether they received special school accommodations, such as having a current Individualized Educational Plan (IEP) or 504 Plan. If they reported that they had an IEP or 504 Plan, they were classified as having a disability. Disability status was coded as 0 and non-disability status was coded as 1. For sexual orientation, adolescents were asked to select one of the following options: straight, lesbian, gay, bisexual, questioning, or other. Sexual minority status was dichotomized; sexual minority status was coded as = 0 and straight was coded as 1.

### 2.3. Procedures

IRB approval (Protocol Number: #MW081619PSY) was obtained from the second author’s university and all APA ethical standards were followed during the duration of the study. Twenty high schools were selected from a random list of 150 schools. A recruitment email or phone call was made to school principals, with eight responding positively to the email, two responding that they had other commitments, and the rest never responded. Meetings were then conducted among school principals, teachers, and research personnel. The purpose of the meeting was to introduce school principals and teachers to the study and explain the procedures for data collection. Afterward, classroom announcements were made to 9th grade classrooms and parental permission slips were disseminated. Approximately 2636 parental/guardian permission slips were distributed to adolescents, and 2506 parents/guardians agreed to allow their child to participate, 91 declined, and 39 were never returned. During data collection, which took place in Fall 2019, adolescents provided their assent; three declined to participate.

### 2.4. Data Analyses

To answer the two research questions, a structural regression model (estimator = MLR) was conducted using *Mplus* [69]. Paths were added from all sexting behaviors (i.e., consensual sexting, non-consensual sexting, pressured sexting) to depressive symptoms and non-suicidal self-harm. Paths were also added from demographic variables (i.e., gender, ethnicity, disability, sexual minority status) to sexting behaviors, depressive symptoms, and non-suicidal self-harm. Two-way interactions were included between demographic variables and sexting behaviors, resulting in interactions between consensual sexting and gender, consensual sexting and ethnicity, consensual sexting and disability, consensual sexting and sexual minority status, non-consensual sexting and gender, non-consensual sexting and ethnicity, non-consensual sexting and disability, non-consensual sexting and sexual minority status, pressured sexting and gender, pressured sexting and ethnicity, pressured sexting and disability, and pressured sexting and sexual minority status. We did not consider age differences in this study because adolescents were typically around the age of 15.

Goodness of fit indices were also assessed, including the comparative fit index (CFI), the Tucker–Lewis Index (TLI), the root mean square error of approximation (RMSEA), and the standardized root mean square residence (SRMR). Adequate fit was determined by CFI and TLI values above 0.95, values less than 0.05 for RMSEA, and values less than 0.08 for SRMR [70]. The Interaction program was used to examine significant interactions to provide the significance of the slopes.

## 3. Results

### 3.1. Preliminary Analyses

Descriptive statistics for the sexting scales are reported in Table 1.

Bivariate correlations were conducted among all the study’s variables (see Table 2). The correlations revealed that consensual sexting was related positively to non-consensual sexting and pressured sexting, but not to depressive symptoms or non-suicidal self-harm. Non-consensual sexting was associated positively to pressured sexting, depressive symptoms, and non-suicidal self-harm. Pressured sexting was related positively to depressive symptoms and non-suicidal self-harm. Depressive symptoms were related positively to non-suicidal self-harm.

### 3.2. Associations between Sexting and Depression and Non-Suicidal Self-Harm

The structural regression model (Table 3) had adequate fit, χ^2^ = 1031.19, df = 894, *p* < 0.001, CFI = 0.99, TLI = 0.98, RMSEA = 0.04, SRMR = 0.05. Regarding RQ1, it was found that consensual sexting was not significantly related to either depressive symptoms (β = 0.03, *p* = 0.886) or non-suicidal self-harm (β = 0.08, *p* = 0.763.). Non-consensual sexting was positively associated with depressive symptoms (β = 0.27, *p* < 0.006) and non-suicidal self-harm (β = 0.23, *p* < 0.024). Pressured sexting was also positively related to depressive symptoms (β = 0.30, *p* < 0.001) and non-suicidal self-harm (β = 0.27, *p* < 0.003).

Regarding RQ2, the results revealed that boys reported engaged in more non-consensual sexting when compared to girls (β = −0.19, *p* < 0.029), whereas girls reported experiencing pressured sexting more so than boys (β = 0.22, *p* < 0.031). Sexual minorities indicated that they engaged in more consensual sexting when compared to non-sexual minorities (β = −0.21, *p* < 0.026). No other differences in sexting were found for the demographic variables. There were no gender, ethnic, disability, or sexual minority status differences in depressive symptoms or non-suicidal self-harm.

For depressive symptoms, significant two-way interactions were found between gender and pressured sexting, ethnicity and pressured sexting, and sexual minority and pressured sexting. For non-suicidal self-harm, two-way interactions were found between gender and pressured sexting, ethnicity and pressured sexting, and sexual minority and pressured sexting. Examining interactions further revealed that girls, white adolescents, and straight adolescents who experienced pressured sexting reported greater depressive symptoms and non-suicidal self-harm. No significant interactions were found for consensual sexting and non-consensual sexting or for disability status (see Table 3).

## 4. Discussion

The aims of the present study were to investigate a) the relationships between three types of sexting behaviors, namely consensual, non-consensual, pressured sexting, and depressive symptoms and non-suicidal self-harm and b) the moderating effect of adolescent demographic variables (i.e., gender, ethnicity, disability, sexual minority) in the associations among three types of sexting, depressive symptoms, and non-suicidal self-harm. The research addresses gaps in the literature on the associations between sexting behavior and psychological health by taking into account various types of sexting and the potential impact of demographic variables on these relationships. The findings might inform the development of programs aimed at reducing sexting risks and the negative outcomes by being sensitive to the various types of sexting and adolescents’ diversity.

Regarding the first research question, we found that consensual sexting was not associated with depressive symptoms but non-consensual and pressured sexting were each positively related. This finding is difficult to compare with prior research because of the lack of research that investigated all three types of sexting in relation to depressive symptoms in one analysis. In comparison to prior work that used global items to measure sexting, our finding is somewhat aligned with studies that revealed a positive correlation between sexting and depression [33,46] but also with other studies that did not [43,45]. Comparing our findings to prior research that specified a particular type of sexting in relation to depressive symptoms, our findings contrast with several studies which found a significant positive relationship between consensual sexting and depression [31,39,41,47]. Regarding non-consensual sexting, our result is in line with Frankel et al. [39] who found a positive relationship between non-consensual sexting and depression. Our findings regarding pressured sexting are partially in accordance with Englander [38] who found that pressured sexting and not-pressured sexting were positively correlated with depressive symptoms. The only study that used a very similar instrument for measuring sexting was from Morelli et al. [27]. The authors did not find an association between sexting and psychological distress (e.g., depression). There are a few differences which might explain the divergent results. First, the participants were much younger in the present study (*M*_age_ =15.1 vs. *M*_age_ = 20.8). Young adults might be more capable of coping with stress resulting from aggravated sexting. Second, different measures were used for psychological health issues in both studies. Third, Morelli and colleagues [27] did not analyze the relationship between sexting and psychological health by considering consensual, non-consensual, and pressured sexting separately but instead included three groups of sexters based on the amount of sexting. In addition, this typology included items on sending and receiving sexts and not only sending sexts as in the present study. More research is needed to understand whether methodological differences in measuring sexting might explain the varying findings.

As for depressive symptoms, we found the same pattern for the associations between sexting and non-suicidal self-harm. More specifically, non-consensual and pressured sexting were both significantly and positively related to non-suicidal self-harm but consensual sexting was not. This finding is partially in line with research based on using global sexting items [18], research that found a significant relationship between pressured and not-pressured sexting and digital self-harm [38], and research that showed a positive correlation between consensual and non-consensual sexting and self-harm [39].

In general, the present study suggests that consensual sexting might not be correlated with psychological problems in the same way that non-consensual and pressured sexting is. We propose that the non-mutuality of aggravated sexting might negatively harm adolescents’ psychological well-being and thus, disrupt normal functioning [12,33,34,35]. More research is needed to understand whether a loss of reputation, experiences of blame, and feeling helpless might further strengthen the negative relationship between aggravated sexting and psychological health. This information might help to design intervention measures to support victims of aggravated sexting and reduce potential effects on their psychological health. The present study also indicates the need to take into account all three forms of sexting in one analysis to partial out the potential effects of different forms of sexting on psychological health.

Concerning the second research question, findings revealed that sexual minorities engaged in greater consensual sexting than non-sexual minorities, boys engaged in greater non-consensual sexting when compared to girls, and girls experienced greater pressured sexting in comparison to boys. The literature is mixed on gender differences in sexting behaviors [30,31,47,49,50,51,53], but it often stresses that girls are more affected by pressured sexting and non-consensual sexting [71]. The available literature indicates that sexual minority adolescents engaged in greater sexting behaviors than non-sexual minority adolescents [14,31,65], with the present research specifying a specific type of sexting behavior, particularly consensual sexting. It might be likely that the online environment helps to build intimacy among sexual minority adolescents and protects against sexual stigmas, making sexual minority adolescents freer to communicate sexually with each other [19,72]. Boys were more likely to forward sexual photos than girls and finding that boys engaged in more non-consensual sexting seems aligned with the current literature [54]. Furthermore, girls indicated that they were asked more often for sexual photos than boys [54]. Thus, finding that girls experienced greater pressured sexting than boys is consistent with the previous literature. Apparently, traditional gender roles and gendered sexual norms of male activity and aggressiveness versus female passivity are reflected in adolescent sexting behaviors.

Ethnicity and disability status were not associated with any type of sexting. Such findings are difficult to reconcile with the lack of existing research on these topics. Although the current literature compares ethnic minorities, there is a lack of research among adolescents. In one study among college students, Benotsch et al. [20] found that white students were more likely to engage in sexting than non-white students. Our results do not confirm such findings among adolescents in our sample. No studies have examined the role of disability status in sexting behaviors among adolescents. Thus, the present study is one of the first and indicates similar patterns of sexting behaviors among disabled and non-disabled adolescents.

Moderation effects were found for pressured sexting only. The relationship between depressive symptoms and pressured sexting was stronger for girls (vs. boys), non-ethnic minority adolescents (vs. ethnic minority), and non-sexual minority adolescents (vs. sexual minority), with similar patterns found for non-suicidal self-harm. Pressured sexting might have an increased risk of having sexual images and chats shared with others, which could harm the victim’s reputation and increase emotional impacts [33,43]. As the sexual double standard is still prevalent, it makes sense that girls are more vulnerable than boys when their sexual reputation is threatened by circulating sexting images. There was no moderating effect of disability status found in this study. The lack of research on disability status and sexting makes it difficult to compare the current findings with the literature. Overall, this finding indicates that the negative outcomes of sexting are not influenced by adolescents’ disability status. Such a finding might further highlight how disabled and non-disabled adolescents might equally utilize information and communication technologies for sexual exploration, and that the negative impacts are not uniquely associated with either type of adolescent.

## 5. Limitations and Future Directions

The present study has some limitations and future research might help to address such limitations. First, the data were cross-sectional, and therefore, temporal inferences cannot be made. There is also no possibility to examine temporal or causal ordering among variables. Thus, future research should incorporate longitudinal designs to understand the relationships among types of sexting, depressive symptoms, and non-suicidal self-harm. In addition, the longitudinal design might make it possible to determine the ordering of the variables examined in this study. The used scales for measuring sexting types differed regarding the wording and response options which limits the possibility of comparison (e.g., frequency rates). Follow-up research should use one scale that measures all different types of sexting in a similar way. In addition, pressured sexting was measured only by two items which is problematic in terms of reliability and validity. Follow-up research should include scales with more items to overcome this methodological shortcoming. The variable of ethnic minority was dichotomized into non-ethnic minority and ethnic minority. Such a procedure was utilized because there were much more non-ethnic minority adolescents in the study. Follow-up research should aim to recruit more ethnic minority adolescents to better examine differences across different ethnic minorities and compare such differences to non-ethnic minority adolescents. Similarly, we combined all sexual minority adolescents and adolescents with disability status into one group each. A fruitful direction in the literature would be to sample enough minority adolescents to examine differences among lesbian, gay, bisexual, queer, and questioning adolescents or adolescents with different disabilities. The same holds true for the coverage of gender diversity: future studies should aim at oversampling gender-diverse adolescents (e.g., transgender, intergender, non-binary, gender-queer) to explore their involvement in sexting that seems to differ from that of non-gender-minority youth at least in terms of pressured sexting [9]. Although not investigated in this study, future research should examine the interaction of gender, ethnicity, disability, and sexual minority status to investigate the intersection of different identities on adolescents’ sexting behaviors and outcomes.

## 6. Conclusions and Implications

In both public and academic discourses, adolescent sexting has been framed as either a problematic and harmful behavior associated with severe negative outcomes such as depression, self-harm, or even suicide (deviance discourse) or a normal and age-normative behavior expressing intimacy and sexual interest among peers in the digital age (normalcy discourse; [4]). Acknowledging that adolescent sexting is a complex phenomenon including both consensual benevolent behaviors (consensual sexting) and non-consensual aggressive behaviors (aggravated sexting with the two subtypes of non-consensual sexting and pressured sexting) resolves this dispute. While consensual sexting can be regarded as a statistically and developmentally normal expression of adolescent sexuality unrelated to negative effects such as depression or self-harm, aggravated sexting can be regarded as a new digital type of aggressive and harmful behavior that is linked to negative psychological outcomes in victims.

To stress these important differences supported by a growing body of literature [40,73], including the present study, it would be helpful to use more appropriate labels in the future. Just as we do not refer to sexual assault or rape as “aggravated sex”, we should not talk about “aggravated sexting” when sexually suggestive or explicit photos or videos are taken, obtained, exchanged, and/or disseminated against the will of the victim. This aggressive and gendered behavior is more clearly addressed in terms of sexual coercion or assault and sexual harassment when it occurs among individual peers. Furthermore, the circulating of sexually suggestive or explicit pictures against the will of the victims among groups of friends, in whole school classes or schools is more clearly addressed in terms of sexual and gendered bullying to point to the fact that it goes beyond dyadic aggression but involves many perpetrators and bystanders plus an institutional environment (e.g., school setting) that fails to protect victims [74,75].

The correct labeling of the phenomena is a first step to improved prevention of sexting harms. Instead of dramatizing or moralizing the sexual content of adolescent sexting and blaming victims for their involvement in sexual behaviors, prevention messages and programs should better focus on the core issue of consensual behavior versus sexual assault and harassment or bullying when it comes to self-produced explicit images. This clarification would also help to avoid prevalent victim blaming [74] and to stress the responsibility of the educational system to provide school settings free of sexual and gendered aggression and violence both offline and online.

While sexting behaviors have been the subject of a growing body of literature over the last few years, evidence-based prevention messages and programs are scarce. Based on the current study and previous research, the following messages for adolescents, parents, and educators seem to be relevant and evidence-based when it comes to the prevention of sexting harms:

(1) *Consensual sexting*: In the digital age, young people’s intimate communication takes place offline and online. While intimate communication and relationships come along with risks, they are part of growing up and also provide important resources. Hence, sexting can be understood as a normal sexual behavior widespread among adults that youth are growing into. Acknowledging the normalcy of sexting is an important message for prevention of sexting harms because youth will only share concerns, worries, or problems around their sexting experiences if they can be sure not to be judged, stigmatized, or punished by parents, educators, or peers for their sexting involvement. However, stressing that consensual sexting is a legitimate and normal behavior among adolescents not to be stigmatized or moralized does not mean to promote sexting as something necessary, mandatory, or cool per se. Just as any other sexual behavior, it is a matter of personal preference and appropriate context. Prevention messages can include empirical evidence regarding sexting prevalence in different age groups as well as reasons and relationship contexts that might motivate young people for or against sexting. Additionally, pragmatic tips on “safer sexting” can be shared (e.g., on how to use photos that are not identifiable or where to get help immediately if something stressful occurs around sexting). It also should be clarified that sexting as intimate communication always requires explicit consent and privacy.
(2) *Pressured sexting*: As with all sexual behaviors, the issue of consent is crucial but not always clear-cut. Young and inexperienced people might be particularly vulnerable to peer-pressure and seemingly agree to behavior they do not want just to receive attention, appreciation, or win friends. Hence, it is important to empower youth and particularly girls as well as gender and sexual minority youth to clearly reject behaviors they do not really feel comfortable with. At the same time, it is important to sensitize youth and particularly boys to the necessity to obtain explicit and enthusiastic consent before they initiate sexting. Gendered aspects of pressured sexting should be integrated in prevention messages, e.g., in terms of a reflection of traditional gender roles that expect girls to present themselves in a sexy way or that expect from boys sexual conquests—expectations that easily translate into pressured sexting and gendered power (abuse) relations [55].
(3) *Non-consensual sexting*: To violate the privacy and confidentiality of intimate communication such as sexting by sharing and disseminating another person’s sext without their knowledge or consent is unethical and also illegal in most jurisdictions. Hence, it is important to sensitize youth to the fact that sharing other people’s sexts is neither normal nor “funny” as many believe [76] but a seldom, highly unethical, and even illegal behavior. Legal consequences should be explained as well as the severe negative effects on victims of sexual image-based bullying. To foster empathy and solidarity with bullying victims it is important to de-stigmatize sexting as suggested above. Otherwise, the perpetrators will continue to excuse their behavior by blaming the victims for their ostensibly questionable sexting behavior (“if they create nude selfies it’s their fault when the images are shared”). To address non-consensual sharing of sexts as image-based bullying also helps to develop target-specific prevention messages for bullies and for the often large group of bystanders who could and should intervene as soon as they hear about a sext that is circulated within a school, class, or group of friends.

To further elaborate evidence-based prevention messages and implement them in local, national, and international prevention programs is important to better protect youth from sexting harms. Here, a participatory approach including adolescents voices is recommended [13,77]. Sexting harm prevention programs should go beyond prevention messages that target teens, though. They must include institutional support systems, intervention plans, school collaborations with parent organizations, professional anti-violence counseling centers, and ultimately, law enforcement.

## Figures and Tables

**Table 1:** Descriptive statistics of sexting types by demographic variables.

	Consensual Sexting	Non-Consensual Sexting	Pressured Sexting
	M (SD)	M (SD)	M (SD)
All	5.69 (0.82)	3.83 (0.66)	3.96 (0.70)
*Gender*			
Male	5.69 (0.83)	3.98 (0.66)	3.88 (0.63)
Female	5.71 (0.86)	3.71 (0.59)	4.01 (0.70)
*Ethnicity*			
Minority	5.66 (0.88)	3.82 (0.61)	3.90 (0.68)
White	5.70 (0.81)	3.86 (0.60)	3.93 (0.69)
*Disability Status*			
Disability	5.60 (0.78)	3.84 (0.63)	3.92 (0.71)
Non-Disability	5.66 (0.80)	3.86 (0.68)	3.94 (0.74)
*Sexual Orientation*			
Sexual Minority	5.82 (0.92)	3.80 (0.71)	3.93 (0.72)
Straight	5.63 (0.81)	3.82 (0.76)	3.97 (0.73)

Note. Due to the different response options for the scales measuring sexting types, the mean values of consensual sexting (1–7) and aggravated sexting (1–5) are not comparable.

**Table 2:** Correlations among variables.

Variables	1	2	3	4	5
Consensual Sexting	---				
Non-Consensual Sexting	0.25 **	---			
Pressured Sexting	0.19 *	0.41 ***	---		
Depressive Symptoms	0.10	0.30 ***	0.30 ***	---	
Non-Suicidal Self-Harm	0.09	0.29 ***	0.33 ***	0.44 ***	---

Note. * *p* < 0.05. ** *p* < 0.01. *** *p* < 0.001.

**Table 3:** Structural regression model results.

	**Consensual Sexting**			**Non-Consensual Sexting**			**Pressured Sexting**		
	*β*	*SE*	*p*	*β*	*SE*	*p*	*β*	*SE*	*p*
Gender _female_	0.10	0.01	0.451	−0.19	0.04	0.029	0.22	0.03	0.031
Ethnicity _white_	0.07	0.01	0.576	0.13	0.03	0.403	0.13	0.02	0.426
Disability _non-disability status_	0.16	0.03	0.313	0.10	0.02	0.379	0.09	0.01	0.391
Sexual Minority _straight_	−0.21	0.05	0.026	0.16	0.02	0.201	0.13	0.02	0.376
	**Depressive Symptoms**			**Non-Suicidal Self-Harm**					
	*β*	*SE*	*p*	*β*	*SE*	*p*			
Gender _female_	0.04	0.01	0.801	0.12	0.03	0.444			
Ethnicity _white_	−0.10	0.02	0.403	0.06	0.01	0.799			
Disability _non-disability status_	−0.03	0.01	0.777	−0.08	0.02	0.413			
Sexual Minority _straight_	−0.14	0.03	0.359	−0.14	0.03	0.367			
Consensual Sexting (CS)	0.03	0.02	0.886	0.08	0.02	0.763			
Non-Consensual Sexting (NCS)	0.27	0.06	0.024	0.23	0.05	0.024			
Pressured Sexting (PS)	0.30	0.10	0.001	0.27	0.08	0.003			
Gender ×CS	0.03	0.01	0.726	0.04	0.01	0.813			
Gender × NCS	0.10	0.02	0.451	0.10	0.02	0.449			
Gender × PS	0.20	0.05	0.019	0.18	0.04	0.032			
Ethnicity × CS	0.01	0.01	0.834	0.02	0.01	0.841			
Ethnicity × NCS	0.11	0.02	0.463	0.09	0.02	0.479			
Ethnicity × PS	0.15	0.03	0.039	0.19	0.04	0.021			
Disability × CS	−0.06	0.02	0.801	−0.10	0.03	0.487			
Disability × NCS	−0.03	0.01	0.813	−0.02	0.01	0.801			
Disability × PS	−0.11	0.02	0.460	−0.10	0.03	0.453			
Sexual Minority × CS	0.10	0.02	0.448	0.09	0.02	0.439			
Sexual Minority × NCS	0.10	0.02	0.442	0.01	0.01	0.831			
Sexual Minority × PS	0.19	0.04	0.018	0.19	0.04	0.020			

Note. Gender was coded as 0 for boys and 1 for girls. Ethnicity was coded as 0 for minority status and 1 for White. Disability status was coded as 0 and non-disability status was coded as 1. Sexual minority status was dichotomized, with 0 = Sexual minority status and 1 = Straight.

## Data Availability

The data presented in this study are available on request from the corresponding author. The data are not publicly available due to legal and privacy issues.
